# Evaluating the effect of a new myopia control spectacle lens among children in Israel: 24-month results

**DOI:** 10.1038/s41433-026-04455-8

**Published:** 2026-05-09

**Authors:** Yuval Cohen, Otzem Chassid, Laura Benhaim-Sitbon, Shirel Ratzon, Dana Gotthilf-Nezri, Atalia Weiss, Noam Baran, Nir Erdinest, Yair Morad

**Affiliations:** 1 Department of Ophthalmology, Ziv Medical Center, Safed, Israel; 2 Azrieli Faculty of Medicine, Bar-Ilan University, Safed, Israel; 3 School of Optometry and Vision Sciences, Faculty of Life Sciences, Bar-Ilan University, Ramat-Gan, Israel; 4 Moar Clinic, Petach Tikva, Israel; 5 Shamir Optical Industry, Research and Clinical Department, Shamir, Israel; 6 Hadassah Medical Center, Ophthalmology, Jerusalem, Jerusalem District Israel; 7 Department of Ophthalmology, Shamir Medical Center, Be’er Ya’akov, Israel; 8 Sackler Faculty of Medicine, Tel-Aviv University, Tel Aviv, Israel

**Keywords:** Outcomes research, Physiology

## Abstract

**Aim:**

To investigate the effectiveness of a novel spectacle lens designed to slow the progression of myopia in children.

**Methods:**

In this prospective clinical trial, children aged 6–13 years (*N* = 126) with spherical equivalent (SER) refractive errors of –0.5 to –6.25 dioptres (D) were randomised into either the Shamir Myopia Control (SMC) lens group or single-vision spectacle lenses (SVL) groups. Outcome measures were changes in axial length (AL), SER, and subjective rating of visual experience (SVE).

**Results:**

After 24 months, AL elongation in the SMC group (N:38) was slowed by 33%, while SER progression was slowed by 26% compared to SVL (N:37, *p* < 0.05). In the SMC younger subgroup (6–10 years), AL elongation was slowed by 0.28 mm (44%, *p* < 0.001) while SER progression was slowed by 0.53 D (43%, *p* < 0.05). In the SMC subgroup with 2 myopic parents, AL elongation was slowed by 0.18 mm (*p* < 0.001) while SER progression was slowed by 0.55 D (43%, *p* < 0.001). SVE reported in a 24-month questionnaire revealed no difference between the SMC and SVL groups. The average daily wearing hours reported in 24 months were similar in the SMC and SVL groups: 14.7 and 14.9 h, respectively.

**Conclusion:**

After 24 months of continuous wear, the SMC lens was proven to be effective in slowing the progression of myopia, especially in children younger than 10 years and in children with two myopic parents. SVE and the prolonged daily use reported by the SMC group indicated good lens tolerability.

## Introduction

Myopia is a global public health concern with a steadily increasing prevalence and a progressively earlier age of onset [1–3]. Beyond the evident impacts on quality of life and the economic burden of optical solutions, myopia is linked to a higher risk of various health issues, including retinal detachment, myopic maculopathy, early cataract development, and strabismus [4–8]. The severity of these complications correlates with the degree of myopia, underscoring the importance of curbing its progression.

In recent years, treatment options to slow myopia progression have been utilised worldwide. One method to achieve that goal is to induce myopic defocus on the peripheral retina using special spectacle lenses. It is postulated that the induction of peripheral retinal defocus induces retinal signals that retard axial length elongation, while the central vision is intact and fully corrected [9–12].

The Shamir Myopia Control lens is a newly designed myopia defocus lens, manufactured by Shamir Optical Industry Ltd. (Kibbutz Shamir, Israel), implementing defocus in a unique back surface design. Utilising Shamir Free Form technology, the lens features a smooth and clear design without any visible patterns on the lens surface. The defocus design is unique in being non-circular, but rather a U-shaped pattern that creates a clear central vertical canal to achieve minimal disturbance to the central and lower visual plane, while inducing peripheral defocus in the lens periphery. In this study, we report the effect of 24 months of wearing these lenses compared to single-vision conventional lenses in slowing myopia progression in children, as reflected by axial length elongation and spherical equivalence progression. The results of the first 12 months of this trial were reported elsewhere [13].

## Methods

This trial was conducted according to the applicable local regulations and the GCP. All essential documents were reviewed and approved by the Ethics Committee prior to the trial. Any amendments to these documents were reviewed and approved by the Ethics Committee before implementation in the trial. The trial was registered at the NIH (ClinicalTrials.gov Identifier: NCT05477329). Written informed consent was obtained from all participants’ legal guardians.

### Spectacle lens design

**Fig. 1:**
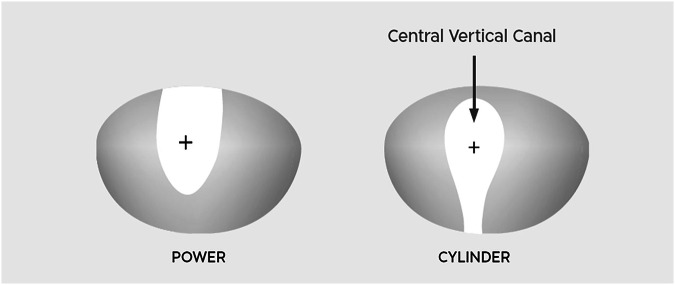
SMC lens design scheme—central vertical canal—addition and cylinder maps.

The SMC lens (Fig. 1) features a “Central Vertical Canal” that corrects distance vision and a peripheral zone that induces myopic defocus (peripheral defocus). The central vertical canal is 10 mm wide horizontally and extends vertically to the lens periphery. The peripheral defocus outside the central vertical canal gradually increases from 0.5 D at the edge of the canal to 3.00 D at 17.5 mm horizontally and 1.50 D at 16 mm inferiorly. This design maintains good-quality vision for distances and downgaze while allowing a comfortable posture on near downgaze.

The horizontal myopic defocus structure enhances lens aesthetics and reduces the lens thickness in the periphery known as the “cut-in effect”. This aims to provide a more natural look for myopic children, supporting all-day wear for effective myopia management.

The study methods are described in detail elsewhere [13]. Briefly, this was a controlled, randomised, double-masked trial involving 126 myopic children (with SER of −0.5 D to −6.25 D and astigmatism of up to 1.5 D) aged 6–13 with no ocular abnormalities. Children were divided into two groups: those wearing the SMC myopia control spectacle lens and those wearing a standard single-vision spectacle lens. Participants were followed for 24 months, with follow-up visits every 6 months. Baseline measurements included objective and subjective refraction under cycloplegia, axial length (AL) measurements, and functional vision tests such as visual acuity, Titmus Test and visual fields on confrontation. Children were required to wear their assigned lenses throughout the day, and any significant changes (greater than 0.5 D) in SER prompted a new lens order. As required by our Ethics Review Board, children with SER progression of more than 1 diopter per year were excluded from the study and referred for treatment with atropine drops.

Subjective visual experiences and lens comfort were assessed through questionnaires during follow-up visits.

### Statistical analysis

The effect of the lenses on progression were analysed using *t* tests and linear mixed models, adjusting for age, gender, baseline refraction/axial length, parental myopia, daily spectacle wear, and treatment duration. A *p* value < 0.05 was considered significant. Analyses were conducted for the entire sample and subgroups by age and parental myopia. Medistat Ltd (Ramat Hachayal, Tel-Aviv, Israel) handled the statistical analysis.

## Results

One hundred twenty-six children with a mean age of 9.92  ± 1.7 years, ranging from 5.7 to 12.8 years, were recruited and randomised to the SMC group (*n* = 65) and control group (*n* = 61) groups. The demographic and ocular characteristics of each group at baseline are shown in Table 1. The parental myopia rate was significantly higher in the control group. The age of myopia diagnosis was 7.18 ± 1.72 years for the SMC group and 6.87 ± 2.06 years for the control group (*p* = 0.37). There was no difference between groups in time spent indoors or outdoors, or in daily activities. Seventy-five children completed 24 months of follow-up (representing 62% of the control group and 57% of the SMC group, respectively). Reasons for dropping out were the aftermath of the October 7, 2023 attack (occurring in N:13, 20% of the SMC group and N:8, 12% of the SVL group), high myopia progression rate (occurring in 6%; N:4, of the SMC group and 7%; N:4, of the SVL group) and visual symptoms (6%; N:3 of the SMC group). Despite this relatively high dropout rate, a statistically significant difference between groups was achieved, indicating a robust clinical effect.

**Table 1:** Demographic and ocular characteristics at baseline.

		SMC group	SVL group	*p* value
Age (years)	*N*	65	61	0.74
	Mean ± SD	9.87 ± 1.71	9.97 ± 1.72	
Gender %	Male/Female	41.5/58.5	54.1/45.9	0.16
Age at Myopia Diagnosis	Mean ± SD	7.18 ± 1.72	6.87 ± 2.06	0.37
Parental Myopia %	None	20	1.64	0.0046
	One parent	38.46	42.62	
	Two parents	38.46	50.82	
	Unknown	3.08	4.92	
Baseline Objective Rx SE (D)	N (eyes)	130	122	0.34
	Mean ± SD	−2.53 ± 1.20	−2.74 ± 1.37	
Baseline Axial Length (mm)	N (eyes)	130	120	0.42
	Mean ± SD	24.27 ± 0.90	24.39 ± 0.78	

Both the study and control group equally complied with wearing the lenses in a satisfying manner with the daily wearing hours averaging 14.70 ± 0.98 in the study group and 14.88 ± 0.78 in the control group. When asked about the subjective rating of their visual experience, such as overall comfort, visual acuity for far, intermediate, and near distance and comfort while walking or engaging in sports activities, both groups were highly satisfied with their glasses, with no statistically significant difference between groups. This effect was maintained during the entire 24 months of the trial.

For the entire cohort, the linear mixed model adjusted means of SER progression were 0.76 D in the study group and 1.03 D in the control group at 24 months, reflecting a 26% reduction (*p* < 0.05), while axial length elongation was 0.33 mm in the study group and 0.50 mm in the control group, indicating a 34% reduction (*p* < 0.01).

**Fig. 2:**
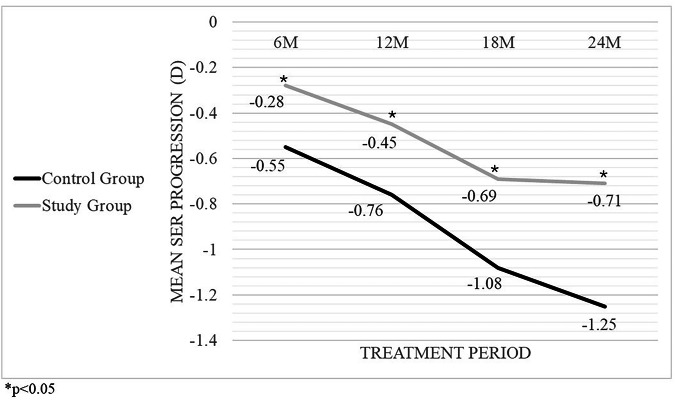
SER progression in children aged 6–10 years.

**Fig. 3:**
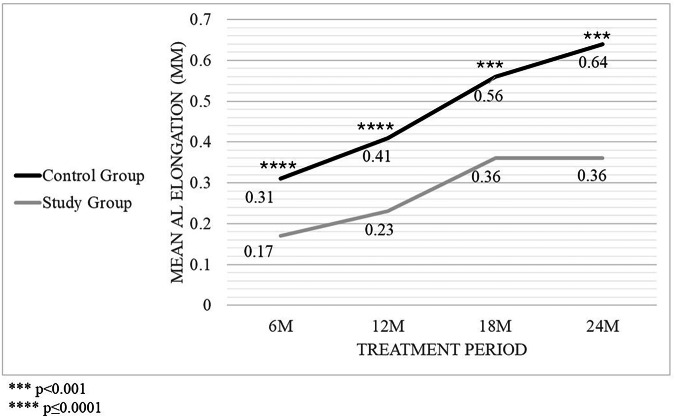
AL elongation in children aged 6–10 years.

Figures 2 and 3 present the progression of SER and AL elongation in children aged 6–10 years in both the study and control groups over a 24-month follow-up period. In this age group, the linear mixed model adjusted means of SER progression in the study group were 0.71 D, while it was 1.25 D in the control group. This difference was statistically significant (*p* < 0.05). Similarly, axial length elongation was 0.64 mm in the control group compared to 0.36 mm in the study group, also showing a statistically significant difference (*p* < 0.001). Wearing glasses resulted in a reduction of SER progression by 43% at the end of the trial compared to controls, with an even higher reduction of 50% during the first 6 months. The effect on axial length elongation was similar: a reduction of 44% overall and 45% during the initial 6 months.

**Fig. 4:**
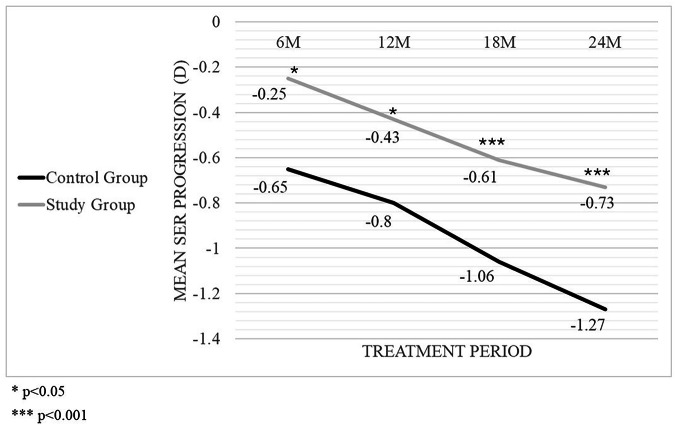
SER progression in children with two myopic parents.

A pronounced effect was also observed in another high-risk group: children with two myopic parents. In this subgroup, linear mixed model adjusted means revealed that wearing the glasses led to a 60% reduction in SER progression during the first 6 months and 43% after two years (Fig. 4), with axial length elongation being slowed by 41% during the first 6 months and 31% at two years. The differences seen in 24 months were highly significant (*p* < 0.001).

In older children aged 10–13 years, both SER progression and axial length elongation were slower in both the study and control groups compared to younger children. According to the linear mixed model adjusted means, the respective figures were 0.82 D and 0.28 mm in the study group as opposed to 0.93 D and 0.42 mm in controls. The difference between the groups was also smaller, with SER being reduced by 24% in 6 months and 12% in 24 months, and axial length elongation reduced by 28% at 6 months and 34% at 24 months. Despite the smaller differences, the 24-months effect in axial length elongation was statistically significant (*p* < 0.05).

## Discussion

In the wake of the myopia epidemic, which is associated with an increased risk of sight-threatening conditions and poses a substantial economic burden on healthcare systems, worldwide myopia management in children has become the standard of care in many countries [14–17]. While atropine treatment initially showed promising results in slowing myopia progression, it continues to face challenges, such as compliance issues, limited access to the medication, potential side effects, and the risk of rebound effects upon discontinuation [18–20]. Recent studies have also questioned the efficacy of low-dose atropine, particularly the 0.01% concentration, which was found to be no more effective than placebo in some trials [10, 21, 22].

Given these limitations, optical treatment options are becoming increasingly appealing, emerging as a key component in the multifaceted approach as they offer potential advantages in terms of ease of use, reduced side effects, and the ability to provide clear vision while simultaneously managing myopia progression [10, 23–25].

In this study, we have shown that the SMC lens is effective in slowing myopia progression as measured by SER and AL elongation. This effect was more pronounced in children with a high risk of accelerated myopia progression, i.e. children aged 6–10 years and children with two myopic parents. In these children, SER progression was slowed by 43% and AL elongation by 44% and 31%, respectively.

Managing myopia in young children poses a distinct challenge, requiring a careful balance between the need for intervention and the practical constraints involved. On one hand, early intervention is crucial as these children are at higher risk of developing high myopia [26]. On the other hand, the primary treatment option of atropine eye drops faces significant hurdles in this age group, as children often resist eye drop administration. Moreover, the need to continue this treatment for several years until around age 12 may lead to compliance issues that can undermine the treatment’s effectiveness [27, 28]. The use of glasses to slow down myopia progression is therefore extremely appealing for this age group.

In our study, SMC lenses had a better myopia control effect in children younger than 10 years compared to the results of some studies conducted with peripheral defocus glasses. In a UK study using MIYOSMART (DIMS) lenses, children under the age of 10 experienced only 15% less axial length elongation compared to controls, as opposed to 57% less progression in children aged 10–15 years [29]. Another study from Germany showed only 11–25% reduction in axial length elongation with DIMS lenses in children younger than 10 years as opposed to 53–60% in older children [23]. In the first report of the 2-year DIMS study done in Hong Kong it was reported that about 80% of children who suffered accelerated myopia progression were aged 8–9 [30]. The authors speculated that retinal profile or peripheral refraction variations might explain the difference.

We are aware of the possible contribution of peripheral refraction variations to the different myopia control effects seen in different age groups, and we would like to speculate that other factors may also play a role: the main difference between the SMC lens and the DIMS lens is the Segmented vs. continuous stimulation they create: DIMS lenses use discrete +3.50 D microlenses in a honeycomb pattern, which creates intermittent myopic defocus zones [31]. In contrast, the SMC lens with its continuous gradient design may provide a sustained, uninterrupted defocus signal across the peripheral retina. Continuous defocus maintains persistent activation of retinal ganglion cells and downstream visual cortex networks, avoiding the intermittent stimulation patterns created by segmented lenses. Younger children’s visual systems, still refining binocular integration and stereopsis, may prioritise these uninterrupted signals for emmetropisation [32].

Another contributing factor may be the difference in ergonomic design between lenses. The SMC lens, with its central clear canal, is designed to enable children to use the glasses continuously, both when looking straight ahead and when looking down during near work.

Children with two myopic parents also showed a significant effect of SMC glasses on myopia progression. Genetic factors undoubtedly play a role in myopia development, whether linked to syndromes like Stickler or Marfan, or through polygenic mechanisms [33]. In addition, environmental factors shared by parents and children alike may also be important [34]. Studies conducted in East Asia reported high rates of parental myopia: in the MIYOSMART study most parents were myopic, and the average refraction of parents in the LAMP Study was also myopic. In contrast, in our study, only 44.4% of children had two myopic parents. This allowed us to examine the impact of this factor on the effectiveness of the glasses, which was found to be significant.

It was well-proven that the effectiveness of myopia-control glasses depends on the duration of use throughout the day. Specifically, it was shown that children using the Aspherical Lenslets glasses for less than 12 h a day demonstrated a reduced myopia control effect compared to children who wore the glasses at least 12 h [35]. In our study, children used the glasses for about 15 h a day on average with minimal variations and no difference between study and control groups. This might contribute to efficient myopia control while using the SMC lenses.

The lack of significant findings in older children (aged 10–13 years) may be due to sample size limitations. The sample size was based on myopia progression data from Western children aged 6–13 years, without accounting for differences between older and younger children. In our study, Israeli children showed a 24-month spherical equivalent refraction (SER) progression of −0.93 D for older children and −1.25 D for younger ones. This smaller difference suggests a larger sample size is needed to achieve statistical significance.

Another significant limitation of our study was the high dropout rate, which was primarily attributed to the geopolitical events that unfolded during the research period. Our study, conducted in Israel, concluded in the aftermath of the October 7, 2023 attack. This unforeseen circumstance significantly impacted the continuity and completion of our research protocols. However, we have no reason to believe that those who remained in the study differ systematically from those who were unable to continue participation. Moreover, the fact that the study retained its statistically significant results despite a 40% dropout rate is a testament to the robustness of our clinical findings, further strengthening the validity of our conclusions. Additionally, because diluted Atropine became an approved and widely used treatment for childhood myopia during the study, our ethical committee decided to exclude children whose myopia progressed by more than 1.00 D per year during the study. This exclusion criterion contributed to study dropout rates, unlike other studies on myopia glasses such as DIMS or aspherical lenslets which did not exclude these children. Visual symptoms caused 6% of the SMC group to drop out within the first few weeks after recruitment. No new cases occurred by the second year, indicating that children tolerated the glasses well after a brief adjustment period. In conclusion, this study demonstrates the efficacy of our novel spectacle lens design in slowing myopia progression, particularly in high-risk populations. The results were especially promising for children under 10 years of age and those with two myopic parents, groups traditionally associated with accelerated myopia progression. These findings not only contribute to our understanding of myopia management but also offer a practical, non-invasive intervention for young children at risk of rapid myopia progression. Future long-term studies are warranted to assess the sustained effects of this lens design and its potential to significantly reduce the burden of high myopia and associated ocular complications in adulthood. Our results suggest that early intervention with these novel spectacle lenses could be a key strategy in mitigating the global myopia epidemic, particularly in high-risk paediatric populations.

## Summary

### What was known before:

- One year follow-up of Shamir Myopia Control lens was published. 10.1016/j.ajo.2023.08.019.

### What this study adds:

- We add the results of the second year follow-up of Myopia Control lens.

## Supplementary information

Clinical Trial Protocol

CONSORT_2025

## Data Availability

The data that support the findings of this study are available from the corresponding author upon reasonable request.
