# Supplementary material for: Evaluating the effect of a new myopia control spectacle lens among children in Israel: 24-month results
Source: Eye (Lond). 2026 May 9;40(11):1649–54. doi: 10.1038/s41433-026-04455-8 (PMC13415571; doi:10.1038/s41433-026-04455-8)
Supplement: Supplementary file 1 — Clinical Trial Protocol [file 41433_2026_4455_MOESM1_ESM.docx]

Evaluating the Effect of a Myopia Control Spectacle Lens Compared to a Single Vision Spectacle Lens Among Children In Israel

Clinical Trial Protocol

Version: 04 Last Updated: 7.6.2021

|  |  |
| --- | --- |
| **Protocol Title** | MCIS2021 |
| **Sponsor** | Shamir Optical Industry Ltd, Israel |
| **Principal Investigator** | Dr. Yuval Cohen, Ophthalmologist |
| **Trial Site** | Maor Ophthalmology Center  Rishon Le Zion  Israel |

Principal Investigator Approval:

| ____________________ | ____________________ | ____________________ |
| --- | --- | --- |
| Principal Investigator  Name | Principal Investigator Signature | Date |

Confidentiality

The content of this document is considered confidential information of the Shamir Group. The sharing of such document is made on strictly confidential basis and may be subject to certain IP rights protection. Please do not disclose, either orally or in writing, this document or any other information included in it, to any other person or third party and do not make any use of it, other than the use permitted by Shamir in advance.

TABLE OF CONTENTS

1. Background………………………………………………………………………………………………………………3
2. Trial Purpose…………………………………………………………………………………………………………….5
3. Trial Design……………………………………………………………………………………………………………….5
4. Trial Product……………………………………………………………………………………………………………..5
5. Enrollment………………………………………………………………………………………………………………..6
6. Trial Plan…………………………………………………………………………………………………………………..7
7. Ethical Issues…………………………………………………………………………………………………………….9
8. Risk Evaluation/Adverse Events………………………………………………………………………………..9
9. Data Management……………………………………………………………………………………………………10
10. Data Analysis……………………………………………………………………………………………………………10
11. References……………………………………………………………………………………………………………….11

| Background |
| --- |

In order to form a clear sharp image when looking at an object, the rays coming from that object should fall on the retina. The retina is a light sensitive tissue located at the inner part of the eye. Myopia (also termed "short-sightedness") is a refractive error of the eye that cause distant objects to appear blurry. This blurry image is formed since rays coming from the distance fall in front of the retina, instead of on the retina. This refractive error is usually corrected by single-vision spectacle lenses or contact lenses. During the progression of myopia, the eye elongates so that its shape becomes more and more "prolate", as opposed to an "oblate" shaped eye.

The eye elongation causes the rays coming from far to fall even more far from the retina so that the myopic refractive error increases and so on. Therefore, the eye length has become a parameter correlated with myopia (myopic eyes tend to have higher eye length than non-myopic eyes).

The prevalence of myopia in the worldwide population has been increasing during the last decades. Half the world population is expected to be myopic by 2050, with 10% having a myopic refractive error of more the -5D^1^. In urban areas of Asia, 80–90% of young adults are myopic and 10–20% have high myopia and the economic burden caused by myopia is also increasing. For example, the annual direct cost of myopia correction for Asian adults has been estimated at US $328 billion/annum. High myopia is associated with complications like glaucoma, macular degeneration, cataract and retinal detachment. High myopes are also affects in other aspects of quality of life due to adverse influences from psychological, cosmetic, practical and financial factors^2^.

The exact mechanism of myopia progression is yet to be fully understood. Researchers around the world have been investigating it for years due to the rise in the prevalence of myopia mentioned above. Among all theories, the *peripheral hyperopic defocus* theory has been extensively investigated as a main component of the myopia progression mechanism, with some clear clinical evidence on both animals and humans. The basis of this theory is that the "prolate" shape of the myopic eye brings about *peripheral hyperopic defocus* (rays coming from objects located in the peripheral visual field fall **after** the retina) which increases eye growth, thereby increases the progression of myopia. There are clinical evidence that defocus induced on retinal areas can influence eye growth^3^. The theory is based on the claim that when the eye detects that rays fall after the peripheral retina (causing *peripheral hyperopic defocus*), it brings about a signal for the eye to elongate in order to bring the photoreceptors closer to the plane of focus.

The amount of peripheral hyperopic defocus can be measured using an open-field autorefractometer which enables measuring the refraction not only in on the visual axis when the child is looking straight ahead but also when the head/eye is rotated so that measurements can be taken from various angles of the eye (therefor various angles on the peripheral retina).

Clinical evidence are now also available for the effect that induced *myopic peripheral defocus* (rays coming from objects located in the peripheral visual field fall **in front of** the peripheral retina) has on slowing down eye growth thereby slowing down the progression of myopia^4^. Also, several kinds of myopia control treatments mentioned below are based on this myopic defocus theory such as orthokeratology, some designs of contact lenses and spectacle lenses. It is known that the hyperopic defocus effect on increasing the progression of myopia is accelerated when the myopic child wears regular single vision lenses for correction of myopia. The prolate shape of the myopic eye along with the induced power of the "minus" single vision lens bring about more peripheral hyperopic defocus which increases the progression of myopia even more. When wearing a myopic defocus spectacle lens, the peripheral hyperopic defocus is significantly decreased or even totally eliminated and turned into a peripheral myopic defocus as shown in figure 1.


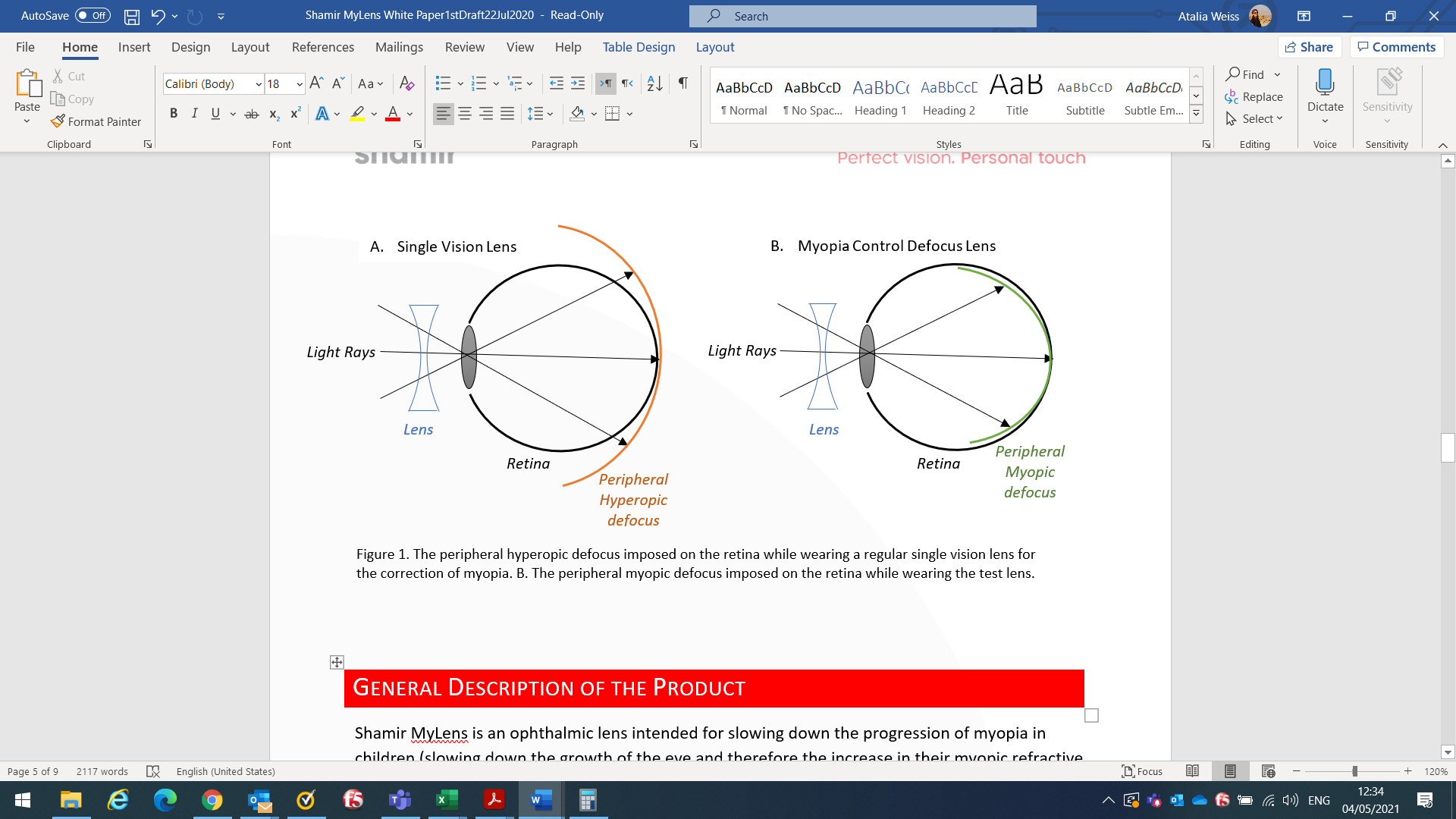


Figure 1. The peripheral hyperopic defocus imposed on the retina while wearing a regular single vision lens for the correction of myopia. B. The peripheral myopic defocus imposed on the retina while wearing the test lens.

The common treatment available for myopia control are Orthokeratology, soft contact lenses, Atropine drops and several kinds of progressive, bifocal or defocus spectacle lenses.

Orthokeratology (OrthoK) is an application of a rigid gas permeable contact lens with a base curve significantly flatter than the corneal curvature to temporarily reduce myopia. OrthoK is used during sleeping time. Clinical evidence point at an effect of up to 40% reduction in myopia progression. Soft contact lenses are also used to control myopia. There are several designs of SCLs available, with the most recent one (MiSight from Coopervision, a dual focus soft contact lens) being effective in up to 60% reduction of myopia and being the first soft contact lenses receiving an FDA approval for controlling myopia progression. Atropine drops for controlling myopia are available in various doses. Low dose Atropine is a common myopia control treatment all over the world including Israel and is available at the Health maintenance organizations in Israel with a deductible, although it's not part of the medical services basket. The low dose Atropine drops are considered to be an effective treatment with no side effects. Some of the spectacle lenses for myopic control have not been clinically evaluated for myopia control at all, some were evaluated and found to be ineffective, and some have declared clinically proven effect of up to 50-60% reduction in myopia progression. A new clinically effective design of spectacle lens is MyoSmart from HOYA (DIMS, defocus incorporated multiple segments). MyoSmart's design comprises a 9mm central optical zone and a 33mm annular zone with multiple 1mm segments having a relative positive power of +3.50 D, thus making the lens appear to have multiple ‘dimples’ in the periphery, although close inspection is required to see them. MyoSmart was clinically tested and found to be effective in up to 60% reduction of myopia compared to a regular single vision lens^5^. Another new clinically effective design of spectacle lens is Stellest by Essilor. Its mechanism is similar to the one of MyoSmart^6^ and its two years results show an effect of 55% in slowing down the progression of myopia^7^. A third spectacle lens is MyoVision by Zeiss. Its mechanism is also based on the defocus concept. An older version of MyoVision was clinically tested and was found to have no significant effect on the progression of myopia^8^. The latest version of MyoVision has not yet been clinically evaluated. Taking into account all available myopia control treatments, a cosmetically appealing, high compliance, low-cost and clinically effective spectacle lens for myopia control is expected to be a well-accepted low-risk and non-invasive treatment.

MyoSmart, Stellest and MyoVision are available to some extent in East Asia and some of them are also in Canada, Australia and Europe. The following table summarizes the advantages that our myopia control test lens is expected to have compared to those 3 designs:

| Vs. | | MyoVision (Zeiss) | Stellest (Essilor) | MyoSmart (HOYA) |
| --- | --- | --- | --- | --- |
| SHAMIR | Efficacy | **Shamir lens is expected to be have a significant effect whereas the effect MyoVision was not clinically proven** | Shamir lens is expected to have a significant effect which will be tested in clinical trials such as this trial. It is yet soon to evaluate whether the effect will be as good as the one of Stellest and MyoSmart. | |
|  | Compliance | **The compliance level with Shamir lens is expected to be good whereas the compliance of MyoVision is still unknown** | **The compliance level with Shamir lens is expected to be better since the peripheral gradual aberrations are expected to create a lower disturbance to vision compared to both Stellest and MyoSmart in which there are many lenslets spread on their entire periphery.** | |
|  | Availability | No advantage is expected. | **Due to a much simple manufacturing technology that is needed to manufacture Shamir lens compared to the complex one needed to manufacture Stellest and MyoSmart, Shamir lens is expected to have a much higher availability to myopic children worldwide in terms of price and manufacturing infrastructure.** | |
|  | Cosmetics | No advantage is expected. | Shamir lens has a better appearance than Stellest and MyoSmart. The many lenslests on those two lesnes are seen on the lens whereas the defocus area on Shamir lens are not seen and the lens is clear (see figure 2 below). | |


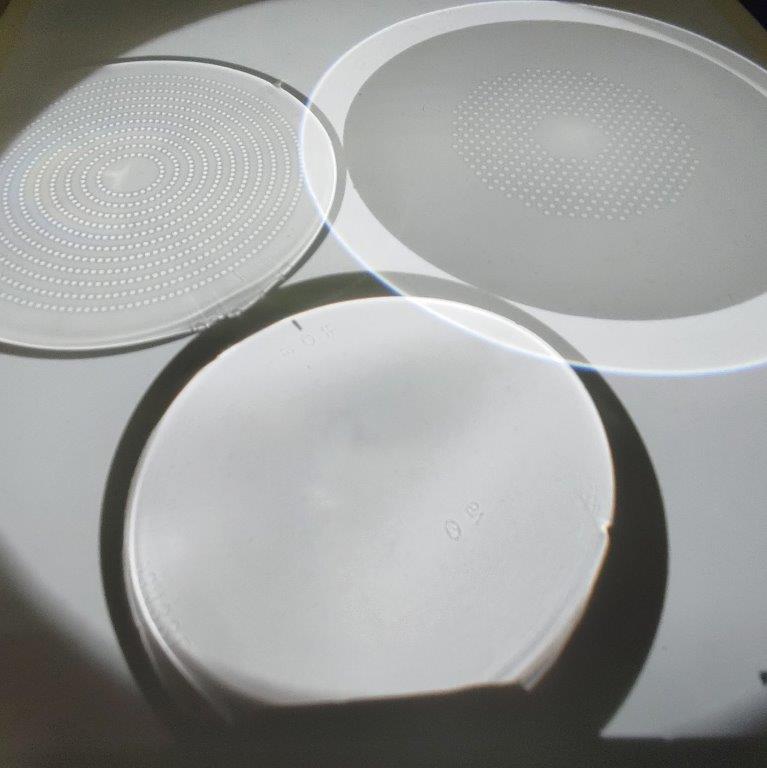


Figure 2. A photo of Shamir lens (the lens at the bottom), Stellest (the lens at the upper left side) and MyoSmart (the lens on the upper right side). It can be seen that Shamir lens is clear whereas Stellest and MyoSmart are not clear due to the many lenslets on their surface.

| Trial Purpose |
| --- |

The purpose of this trial is evaluating the clinical effect of a myopia control spectacle lens compared to a single vision spectacle lens in slowing down the progression of myopia in children living in Israel.

| Trial Design |
| --- |

This will be a controlled, randomized, double-masked trial. The Test group will wear the myopia control spectacle lens and the Control group will wear the single vision spectacle lens.

Primary outcome measure:

The mean change from baseline in objective cycloplegic refractive error in each of the groups (Test and Control)

Secondary outcome measure:

The mean change from baseline in axial length in each of the groups (Test and Control)

| Trial Product |
| --- |

Test lenses: Myopia control design spectacle lens, material: 1.6 High-Index plastic.

Control lenses: Single Vision design spectacle lens, material: 1.6 High-Index plastic.

Both test and control lenses will be designed and manufacture by Shamir Optical Industries Ltd.

Both test and control lenses will be mounted on regular frames.

Test lens

The test lens is manufactured in a standard FreeForm technology (exactly like a standard progressive addition lens which is similar in terms of the graduall power profile induced in both lens). It does not demand any special manufacturing processes.

The test lens should be worn exactly like a standard spectacle lens and it should be worn through all day hours. The fitting of the test lens is done exactly like the fitting of the control lens, according to the manufacturer instructions.

Product Labeling and Accountability

The clinical trial coordinator, who is in charge of the randomization procedures, will be also in charge of labeling the test spectacles per each patient prior to sending the spectacles to the site. The investigator will hold a dispensing log in which there will be a documentation of the product:

- on receival from the manufacturer
- on dispensing to the patient
- on receiving back from the patient if applicable

| Enrollment |
| --- |

**136** participants will be enrolled in this trial (including an estimated dropout rate of ~5%), 68 participants will be randomly allocated to the Test group and 68 participants will be randomly allocated to the Control group.

Sample size calculation

The annual myopia progression rate (in objective refraction) in children in Israel is estimated as 0.6D. The effect estimated is 30% which is a change of 0.18D between the annual myopia progression rate at the test group and the control group. Taking into account a standard deviation of the progression rate of 0.35D (as reported in the scientific literature in western countries), the sample size of each group (test and control) should include 60 children.

Taking into account a drop out rate of 5% and also a certain amount of drop outs during the trial due to high annual myopia progression (as mentioned in the section about terminating the participation in the trial) for which we added 10 more patients, the overall calculated sample size is 136.

Distribution of ages within the sample:

The age range for inclusion in the trial is 6 to 12 years old and the children will be enrolled into two aged groups: young children and older childern. In order to create an equal amount of children within each age group the enrollment will be as follows:

- Young Children: Age ≥6, <9.5: 68 patients
- Older Children: Age ≥9.5, <13: 68 patients

Randomization

Randomization function should be applied separately per each of the two age groups, so that eventually each group will contain an equal number of Test lens wearers and the Control lens wearers.

Randomization function will be applied per each age group by the clinical trial coordinator who will keep the randomization list in a confidential file and will not disclose it to other trial staff members (except for the manufacturing technician at Shamir Optical Industries) until the trial close up unless required in specific cases of trial withdrawals by the investigator.

Inclusion Criteria

- Informed consent form signed and fully comprehended by the parent/legal representative of the child
- Age from 6 to 12 years old
- Both male and female
- Spherical Equivalent refractive error (cycloplegic objective) of -0.50D to -5.00D
- Astigmatism not higher than -1.50D
- Corrected Visual Acuity of not less than 6/7.5 or 20/25
- Willing to wear the trial spectacles for the defined period according the protocol plan

Exclusion Criteria

- Current participation in another clinical trial
- Any general health or ocular health pathology that could affect the treatment
- Allergy or intolerance to cycloplegic eye drops (*name should be stated here*)
- Strabismus
- Amblyopia
- History of ocular injury or ocular surgery
- Previously been treated with any myopia control treatments (Orthokeratology, myopia control spectacle/contact lenses, multifocal lenses, bifocal lenses, atropine)

| Trial Plan |
| --- |

Screening visit **(VISIT 1)**

- Enrollment criteria verification
- Signing informed consent
- Baseline documentation of medical history, parental and siblings myopia, indoor and outdoor activities profile and socioeconomic profile
- Lensmeter measurements of patient's spectacles
- Performing baseline measurements including best corrected VA, objective & subjective refraction under cycloplegia, PR under cycloplegia*, slit lamp examination, functional tests: cover test, titmus test, worth four dot test, axial length** and pupil size measurements
- Frame selection and frame measurements
- Lens order completion and sending to Shamir Optical Industries for manufacturing

Randomization Procedures (following VISIT 1)

Randomization procedures should be performed by the trial coordinator following visit 1. The patient will then be allocated to the test or control group.

Manufacturing Procedures (following VISIT 1)

The manufacturing technician will take care of the manufacturing processes according to the lens type (ib test or control) and the mounting on the frame. The test spectacles will then be sent to the site for delivery.

Delivery visit **(VISIT 2)** performed ~10 days after screening visit

- Trial spectacles delivery
- Subjective questionnaire while wearing the trial spectacles
- Best corrected far and near VA (with the trial spectacles)
- Confrontation visual field test with the trial spectacles
- Giving instructions about wearing the trial spectacles
- Documenting adverse events if relevant

Follow-up visit performed 6 months (±1 week) after the previous visit **(VISITS 3,4,5,6)**

- Documentation of any change medical history since last visit
- Subjective questionnaire regarding the wearing period with the trial spectacles (feedback performing follow-up measurements including best corrected far and near VA, objective & subjective refraction under cycloplegia, PR under cycloplegia*, slit lamp examination, functional tests: cover test, titmus test, worth four dot test, axial length and pupil size measurements
- If the subjective refraction will find a SE (Sphere Equivalent) change from last refraction of at least -0.50D, a new lens order should be made for new lenses, and a delivery visit should be set in order to deliver the new test spectacles. The procedures mentioned above in the delivery visit (visit 2) should then be performed.
- Documenting adverse events if relevant

Additional unscheduled visits

- May be scheduled as needed if special needs occur
- Procedures will be defined by the investigator
- Documenting adverse events if relevant

**Total duration of trial per patient: 24 months.**

**A unique measurement performed using a designated device that measured the refractive error in the peripheral retina and. Duration of measurement – 15 minutes.*

**Axial Length is measured using TOMEY optical biometer OA-2000 (partial coherence interferometry). The measurement is performed without any contact with the patient's eye.

| Ethical Issues |
| --- |

This trial will be conducted according to the applicable local regulations and the GCP. All essential documents will be reviewed and approved by the ethics committee prior to the beginning of the trial. Any amendments of these documents will be reviewed and approved by the ethics committee prior to implantation in the trial.

Informed Consent

The investigator will give the child and their parent/legal guardian a full explanation about the trial and will answer all their question regarding participating in the trial.

The investigator will explain that the child will be able to withdraw from the trial at any stage for any reason.

The child's parent/legal guardian will sign the informed consent form and will receive a signed copy of it.

Data confidentiality

All identification details about the children participating in the trial will be held by the investigator in a secured log file kept at the trial site.

Access to the identification details will not be allowed to any unauthorized party and will not be disclosed in any report/document relating to the trial.

Terminating the participation in the trial

These are the cases in which an early termination of the participation in the trial may occur:

1. Should the myopia progression of the patient will be more than 1.00 D after 1 year or other significant refractive changes (such as more than 0.5[D] cylinder) - the investigator will recommend the patient to discontinue the participation in the trial and to consider using other myopia control treatments such as low dose Atropine drops.
2. Should the far/near VA with the trial spectacles will be less than the best corrected VA of the patient, the investigator will consider recommending the patient to discontinue the participation in the trial.

| Risk Evaluation/Adverse Events |
| --- |

The optical design of the test lens, with the gradual positive peripheral power, induce a certain amount of aberrations in the peripheral areas of the lens. These aberrations were designed such that a good level of compliance will be achieved so that the children will be able to wear the lens.

Adverse events are unlikely to occur, yet it should be mentioned that a child should not wear this lens in case of one/more of the following exist:

- Feeling significant amount of discomfort while wearing the lens such that the safety of the child might be at risk.
- Vestibular disorders

The children participating in the trial will be free to contact the investigator at any time during the wearing period in order to report any unexpected events that might occur. The investigator will decide based on the report whether the child should be excluded from continuing the trial.

Furthermore, it should be noted that a parallel clinical trial is being conducted these days in AIER hospital in China, managed and supervised by Brien Holden Vision Institution (Australia). The trial is similar to our trial, with the same trial product, on a group of 140 children with the same enrollment criteria, including similar trial procedures and trial plan. The trial started at November 2020, and as for now most of the children has worn the test spectacles for a few months with no adverse events reported.

| Data Management |
| --- |

Database

An electronic data base will be held and maintained, into which all the source data will be electronically transferred from the measuring devices, or if not possible, will be typed in and source data (printouts or location of data) will be kept in the patient's file. At any case, all source data (printouts or location of data) should be kept in the patient's file.

The electronic data base will be backed up on a daily basis

Access to data:

The investigator will be able to allow access to data to the Sponsor (without identification data) as per request.

The investigator will be able to allow access to data to the Monitoring staff according to the monitoring plan that will be defined.

| Data Analysis |
| --- |

Data analysis will be performed by a third party. The trial raw data (without the identification data) will be transferred to the third party following the completion of each follow up visit. The third party will then perform the data analysis and produce a report to the sponsor.

Outcome measures analysis:

The effect of the test lens compared to the control lens will be evaluated using the outcome measures mentioned above.

Both the primary outcome measure (the mean change from baseline in objective cycloplegic refractive error) and the secondary outcome measure (the mean change from baseline in axial length) will be calculated per each of the follow up periods mentioned above (6, 12, 18 and 24 months).

The objective cycloplegic refractive error will be calculated in Sphere Equivalent units which is: SPH + CYL /2 wherein the SPH and CYL values will be taken from the objective cycloplegic refraction measurement.

The mean change (and its standard deviation) of each of those parameters per each follow up visit will be calculated as the mean parameter at the follow up visit minus the mean parameter at the baseline visit.

The effect of the lens in terms of progression in those outcome measures will be analyzed using t-test (if data normality exists) and also by using linear mixed models which should be adjusted for age, gender, objective refraction (sphere equivalent), axial length, age of myopia onset, parental myopia, daily time wearing spectacles and time spend outdoors.

The effect we expect to have in the test group is a decrease in one/more of those parameters mean change from baseline (decrease in progression from baseline) compared to the control group. Any such finding that will include a p-value≤0.05 will be defined as statistically significant.

This analysis will be performed for all the sample as a whole and also for sub groups of age (younger and older children as mentioned above).

Differences of baseline parameters between the test and control group

The distribution of the following baseline parameters will be calculated per each of the groups, test and control:

- Objective cycloplegic refractive error (Sphere equivalent)
- Axial length
- Age
- Gender
- daily time spent outdoors
- Parental myopia

Should any significant difference be found between the two groups in any of those parameters, an adjustment should be made in the analysis regarding this parameter.

| References |
| --- |

1. Holden BA, Fricke TR, Wilson DA et al. Global prevalence of myopia and high myopia and temporal trends from 2000 through 2050. Ophthalmology 2016; 123: 1036–1042.
2. Chua S.Y.L., Foster P.J. The Economic and Societal Impact of Myopia and High Myopia. In: Ang M., Wong T. (eds) Updates on Myopia 2020. Springer, Singapore.
3. Smith EL., et al. Effects of Local Myopic Defocus on Refractive Development in Monkeys. Optom Vis Sci. 2013 November ; 90(11): 1176–1186.
4. Wallman J, Winawer J. Homeostasis of eye growth and the question of myopia.Neuron 2004;43:447–68.
5. Bullimore MA, & Richdale K. Myopia Control 2020: Where are we and where are we heading? Ophthalmic Physiol Opt 2020; 40: 254–270.
6. Bao ­J, et ­al. One year myopia control efficacy of spectacle lenses with aspheric lenslets. Br J Ophthalmol 2021;0:1–6.
7. Bao ­J, et ­al. Myopia control with spectacle lenses with aspherical lenslets: a 2-year randomized clinical trial. ARVO abstract 2021.
8. Sankaridurg P, et al. Spectacle Lenses Designed to Reduce Progression of Myopia: 12-Month Results. Optometry and Vision Science, Vol. 87, No. 9, September 2010
